# Increased Release Time of Antibiotics from Bone Allografts through a Novel Biodegradable Coating

**DOI:** 10.1155/2014/459867

**Published:** 2014-06-19

**Authors:** István Hornyák, Edit Madácsi, Pálma Kalugyer, Gabriella Vácz, Dénes B. Horváthy, Miklós Szendrői, Weiping Han, Zsombor Lacza

**Affiliations:** ^1^Institute of Human Physiology and Clinical Experimental Research, Semmelweis University, Tűzoltó utca 37-47, Budapest 1094, Hungary; ^2^Department of Orthopedics, Semmelweis University, Karolina út 27, Budapest 1113, Hungary; ^3^Singapore Bioimaging Consortium, Agency for Science, Technology and Research, Fusionopolis Way, Singapore 138632

## Abstract

The use of bone allografts is contraindicated in septic revision surgery due to the high risk of graft reinfection. Antibiotic release from the graft may solve the problem and these combinations can theoretically be used for prevention or even therapy of infection. The present study investigated whether amoxicillin, ciprofloxacin, and vancomycin alone or in combination with chitosan or alginate are suitable for short-term or long-term bone coating. Human bone allografts were prepared from femoral head and lyophilized. Antibiotic coating was achieved by incubating the grafts in antibiotic solution and freeze-drying again. Two biopolymers chitosan and alginate were used for creating sustained-release implantable coatings and the drug release profile was characterized *in vitro* by spectrophotometry. Using lyophilization with or without chitosan only resulted in short-term release that lasted up to 48 hours. Alginate coating enabled a sustained release that lasted for 8 days with amoxicillin, 28 days with ciprofloxacin coating, and 50 days with vancomycin coating. Using only implantable biodegradable allograft and polymers, a sustained release of antibiotics was achieved with ciprofloxacin and vancomycin for several weeks. Since the calculated daily release of the antibiotic was lower than the recommended IV dose, the calcium alginate coated bone graft can support endoprosthesis revision surgery.

## 1. Introduction

Surgical treatment of musculoskeletal diseases relies increasingly on the long-term implantation of foreign materials such as bone substitutes, endoprosthesis, degradable scaffolds, and plastic components (e.g., polymethyl methacrylate or polyethylene). Since the immune system is not well adapted to fight bacterial infection associated with these foreign materials [1, 2], human allograft seems to be a logical choice as scaffold after suitable preparation [3, 4], although septic complications are becoming a growing concern for the orthopedic community [5, 6]. Infection after total hip replacement is still an unsolved issue; according to a clinical article, infection occurred in 1.7% after primary procedures and in 3.2% after revision procedures [7]. The quantification in another article had different results; in the United States the overall infection burden from 1990 to 2004 of hip arthroplasty was 0.88%; this value increases annually at a rate of close to 5% [8]. Based on another survey, from 145 patients with infection involving total joint arthroplasty subsequent infection occurred in 19% of the cases [9]. Due to the low metabolic rate of bone tissue and the low permeation of the formed bacterial biofilm [10], it is difficult to reach the required local concentration of antibiotics whether it is applied systemically or as a local formulation (e.g., block, sponge, implant, and bead) prepared during surgery [11–14]. In general practice, local treatment is typically applied to support systemic antibiotics and most frequently used drugs include amoxicillin, cephalexin, gentamycin, sulfamethoxazole, ciprofloxacin, and vancomycin applied in cement [15–17], beads [17], and impregnated bone [18–23]. In addition, off-label use of these antibiotics mixed by hand with the carrier bone substitute is often performed when the required antibiotic-carrier combination is not available off the shelf [24]. Whether the applied dose and the release kinetics of such mixtures are optimal or at least adequate for the intended purpose is unknown; however it is still the best way a surgeon can deal with these challenging situations.

The therapeutic goal of local antibiotic use in combination with endoprosthesis can be categorized into three distinct case types: (1) prevention of early infection in a primary prosthesis implantation procedure, (2) inhibiting of infection at aseptic prosthesis revisions where the probability of an already ongoing low-grade infection is high, and (3) treatment of massive infections at septic revisions [25, 26]. These cases pose differing challenges for the antibiotics summarized in Table 1.

It is evident that even if one focuses only on antibiotic bone substitutes several formulations should be available in order to meet these diverging criteria [36]. One way of modifying the release kinetics of drugs in an implantable formulation is to couple the active agent with biodegradable polymers. Two well-known materials, which are frequently used to form biodegradable coatings, are chitosan (Chi) and sodium alginate (Na-Alg) [37, 38]. These biopolymers have been investigated over a wide scale including the preparation of fibers, nanoparticles, and even bone substitutes, thus posing a very low risk of toxicity [39–41]. Chitosan is typically prepared from shrimp-shell chitin with hydrolysis and is only soluble in acidic media. It forms excellent films and coatings and in case it is added to acidic forms of drugs it can slow down release and degradation [42]. Alginate derivatives such as alginic acid or sodium alginate are produced from seaweed species. The main feature of sodium alginate is that it is insoluble in acidic solutions and forms a biodegradable film that can be turned into water insoluble calcium alginate (Ca-Alg), which can act as a barrier for drug coatings. The general view of the surgical community is that local use of antibiotics without any carrier is only effective for the first few days postoperatively; however this view is not supported by reliable experimental data [14, 43]. Theoretically it can be hypothesized that fixation of the antibiotic with physicochemical means such as freeze-drying or embedding in polymer coatings may prolong the release of drugs [43]; however it is unknown if these procedures can meet the requirements detailed in Table 1.

The present study investigated if amoxicillin, ciprofloxacin, or vancomycin dried onto the surface of human bone allografts alone or in combination with Chi or Ca-Alg coating is suitable for preparing an antibiotic implant. We further studied the characteristics (drug release and drug load) of a short-term and a long-term release antibiotic coating* in vitro*.

## 2. Materials and Methods

All chemicals were purchased from Sigma except for vancomycin, which was purchased from Hangzhou APIChem Technology Co., Ltd., China. The HCl salt was used in case of vancomycin and ciprofloxacin; however amoxicillin was used in the trihydrate form. The bone blocks were generous gifts from the West-Hungarian Regional Tissue Bank. Freeze-dried femoral head blocks were cut to 0.05 ± 0.01 g cube-shaped pieces for the experiments.

Antibiotics were used in aqueous solutions with the initial concentrations of 0.1, 1, or 10 mg/mL for preliminary experiments. After evaluating these concentrations, we decided to use a 10 mg/mL starting solution for all further experiments in order to obtain data with low signal-to-noise ratio. The bone graft was placed in 1 mL of the antibiotic solution and the system was incubated at 25°C for 24 hours. Subsequently, the soaked graft was removed from the solution and freezed at −80°C followed by lyophilization for 24 hours using a Labconco Freezone 2.5′ freeze-dryer (soaked preparation). In order to maximize the drug content of the graft an alternative approach was also performed when the grafts were frozen while still being submerged in the antibiotic solution and the whole system was freeze-dried (saturated preparation).

Medium molecular weight Chi was used with a deacetylation grade of 75–85%, and the Chi solution prepared in our experimental set-up contained lactic acid (LA, 90%) with a ratio of 360 *μ*L LA/40 mL 2% Chi. The chitosan-based preparations were prepared by using 1 mL aqueous 2% chitosan solution to dissolve the antibiotic. The bone samples were placed in this solution and incubated at room temperature for 24 hours and frozen and lyophilized afterwards in a similar manner as the saturated preparations.

**Figure 1:**
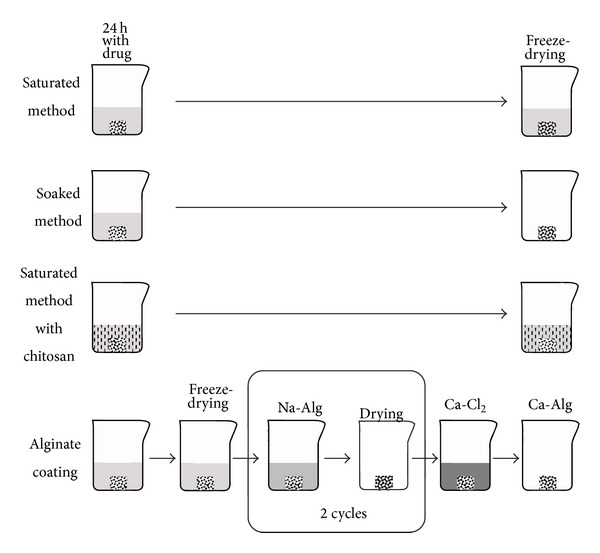
Antibiotic coating methods. As a first step, 50 mg bone allografts were incubated in a 10 mg/mL antibiotic solution for 24 hours. The Chi used for coating was 2 weight %; the alginate was 4 weight %. The final step in each method was lyophilization or drying in an oven resulting in a dry bone allograft which looks the same to the naked eye as a regular uncoated graft.

Alginate-based preparations were created in another way since this polymer exhibits a basic pH and antibiotics typically precipitate in this solution. First, the bone grafts were coated by the saturated freeze-dried method as described above; then a film coating of alginate was created on top of the antibiotic layer. The Na-Alg film was prepared by adding 1 mL 4% Na-Alg solution on the antibiotic coated freeze-dried bone. Then the graft was dried in an oven at 40°C for 4 hours on teflon plates. The process was repeated with the dried coated graft turned upside down; thus the double layer Na-Alg film was formed. Sodium alginate was then converted into calcium alginate by CaCl_2_. The Na-Alg coated bone grafts were placed in a 10% CaCl_2_ solution for exactly 60 seconds, then washed with distilled water, and dried in an oven at 40°C. The methods for preparing the coatings are presented in Figure 1.

The chosen antibiotics (amoxicillin, ciprofloxacin, and vancomycin) have characteristic absorbances in the UV range in aqueous solutions, allowing the use of UV spectroscopy to assess the concentrations with a spectrophotometer. The absorbance-concentration diagrams were plotted using all antibiotics and the linear phase of this diagram was used to calculate the concentration from the absorbances according to the Lambert-Beer law (Table 2).

We conducted preliminary experiments on the possible absorbance of the used polymers; neither Na-Alg nor Chi had detectable spectral peaks where the antibiotics were measured, specifically 229 nm and 275–280 nm, and neither had a significant baseline absorbance even in the maximum possible concentrations used in the present protocol (0.1% Na-Alginate or 0.25% chitosan). In contrast, Ca-Alginate was insoluble in water and the precipitate would have made the specific measurements impossible in case it had been present in the supernatant.

Measurements of release kinetics were performed by incubating each sample separately in 2 mL of water in a 24-well plate at room temperature. We did not work at 37°C to prevent evaporation and we did not use buffers to avoid changes in the solubility and adding possible UV absorbent molecules. Concentration measurements were performed at specified intervals by removing the supernatant for spectroscopy and replenishing with fresh solvent. The frequency of solution changes and the length of the experiments were determined by preliminary experiments and set in ways that optimal kinetic curves could be constructed from the data set. In the case of vancomycin and ciprofloxacin samples were taken on the 1st, 5th, 10th, 15th, 20th, 25th, and 28th day. Vancomycin was also measured on the 50th day. In a separate experiment with Ca-Alg coated amoxicillin grafts, the medium was placed back onto the graft after each measurement in order to evaluate the effect of drug accumulation in the medium. Statistics were carried out using GraphPad Prism 5.0 software. All data were expressed as means ± SEM (*n* = 3) and were analyzed using Student's *t*-test, simple analysis of variance (ANOVA), or 2-way ANOVA with Bonferroni's multiple comparison. Differences were considered significant when *P* < 0.05  (∗), *P* < 0.01  (∗∗), *P* < 0.001  (∗∗∗) (Table 3).

## 3. Results

After preliminary experiments (data not shown), all the three drugs were highly soluble in water and were suitable to be stored at room temperature without any decomposition. The original concentration of the antibiotic solutions used to incubate the bone grafts correlated with the amount of antibiotics on the bone surface as estimated by the total amount of drugs released. We decided to use a 10 mg/mL starting solution for all further experiments in order to obtain data with low signal-to-noise ratio.

**Figure 2:**
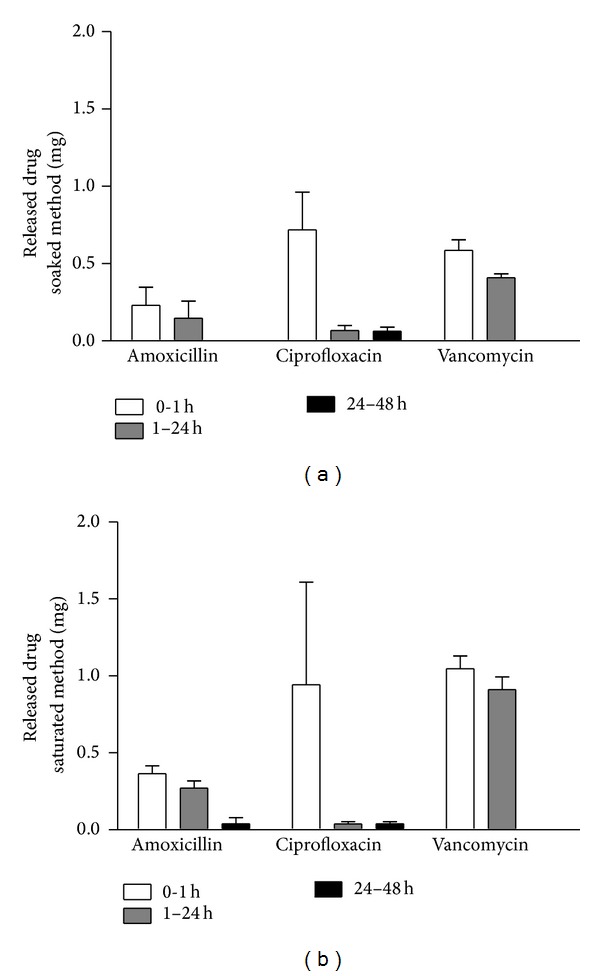
Drug release kinetic of amoxicillin, ciprofloxacin, or vancomycin coating, prepared by the soaked or the saturated method. These procedures are not expected to significantly increase release time, so over 90% of the drug is released within the first day.

Simple freeze-drying of antibiotics on the surface of bone grafts did not result in a sustained release of the compounds. Although minor differences were observed among the three antibiotics, each one is completely released within 48 hours (Figure 2). Maximizing the antibiotic loading with the saturated method did not improve the release kinetics only; the overall amount of antibiotics on the graft was higher (Figure 2(b)). Using a chitosan additive with the antibiotics did not significantly prolong the release of the drugs from the surface.

**Figure 3:**
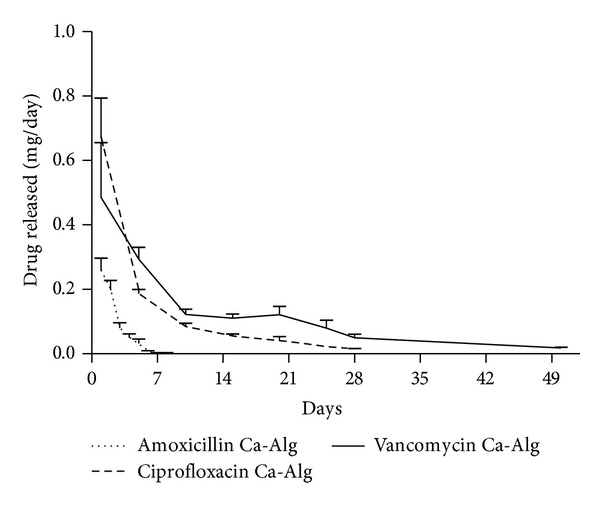
Release profile of amoxicillin, ciprofloxacin, or vancomycin with sustained release Ca-Alg film coating (*n* = 3). Although the coating method was the same in each case, the effective release term was different among the three drugs with amoxicillin lasting up to 8 days and ciprofloxacin up to 28 days while vancomycin reached 50 days.

With the use of a Ca-Alg film layer, it was possible to reach a long-term sustained-release antibiotic coating. Interestingly, the type of antibiotic significantly affected the rate of drug release from the same type of coating. For example, amoxicillin was completely released within 8 days and ciprofloxacin within 28 days while vancomycin exhibited the longest release time with 50 days (Figure 3). The amount of antibiotic released on the first day from Ca-Alg coated allograft was approximately the same as the amount from the antibiotic coated bones which did not contain Ca-Alg (Figure 2). The total quantity of dissolved antibiotics over the 8-, 28-, or 50-day period depending on the respective antibiotic was approximately the same as those without alginate coating. We therefore conclude that the amount of total antibiotic content did not increase; however, the release rate has changed as shown in Figure 3.

In order to test whether the release kinetic is affected by the negative feedback of drug accumulation in the solution, we compared two sets of bone grafts either with release in fresh solvent or with cumulative release into the same medium. Ca-Alg coated amoxicillin was selected for this measurement since it showed a significant change for several days, while the other preparations had too slow or too fast kinetics for this type of measurement. We observed that above the dosage of 0.1 mg/day the release is slightly inhibited by the accumulation of the compound; however this was no issue at lower doses and did not affect the length of the active release period either (Table 3).

To summarize the long-term release experiment, altogether 0.64 ± 0.07 mg amoxicillin was eluted from the surface of 50 mg bone allograft with complete dissolution in 8 days. In case of ciprofloxacin, 1.08 ± 0.11 mg was the total eluted amount within 28 days. Vancomycin had the longest elution time for over 50 days during which 1.66 ± 0.31 mg antibiotic was released in total.

## 4. Discussion

The present study indicated that it is possible to produce antibiotic coating with physicochemical methods. With biopolymers we can modify the release kinetics of antibiotic impregnated bone grafts in order to reach either complete unloading in 48 hours or sustained release for up to 50 days.

A critical limitation in one-stage revision surgery is the extent of bone loss. Ideally, one would perform elaborate bone replacement techniques in order to build a suitable biological base for a new implant; however bone grafts are viewed as contraindicated in these procedures due to the high probability of infection. Impregnation of bone grafts with an antibiotic solution by hand mixing is generally applied in the OR as a preventive measure; however most surgeons would consider this technique inadequate for septic cases. This view is confirmed by data from the present study. Even though the antibiotics were incubated for a day and then freeze-dried onto the bone, the majority of the drug (90%) was released during the first day after placing the graft in water. This release kinetic may be suitable for fighting perioperative infection when the implant may be contaminated during surgery or from the patient's skin through the surgical wound or drainage tubes, but this timeframe is inadequate to eradicate massive infections.

The amount of antibiotics in combination with a bone substitute is a challenge. In most cases the volume of the required bone graft is only determined during surgery and predetermining the required dose is only realistic with large margins. Moreover, the amount of antibiotic which is implanted into a patient is set by the amount of bone graft, as administering the drug follows the “dosing” of the graft. The highly variable spatial conformations add a further degree of freedom to the equation. One would assume that tightly impacted bone chips between a cortical layer and a metal implant have much lower surface for the body fluid to penetrate than a porous block placed into a well bleeding spongiotic area. Our current measurements showed that the negative feedback from the accumulation of the drug in a small volume just marginally affects the release kinetics. Therefore the spatial effect probably plays a limited role in this question. However, it should be noted that our experiment was performed in a laboratory setting and release kinetics with bodily fluids in the presence of metabolizing cells and bacteria will be different. Therefore, due to the uncertainties inherent in this applicable field, it is best to load bone grafts with only a low amount of antibiotics to prevent overdosing. As a comparison in the present study, we estimated the total daily doses potentially released from bone grafts with selected combinations. We applied the femoral head graft as a more or less standard “dose” of bone grafting material frequently applied in orthopedics. For these calculations we estimated the antibiotic elution from a femoral head based on the results from our experiments (Table 4.). In principle we multiplied the antibiotic content based on the femoral head and our bone chip weight ratio (45 g/0.05 g).

Please note that these calculations are based on data gained* in vitro*, so these can only be considered as rough estimates. The calculations show that the implantation of one femoral head coated with any of the analyzed antibiotics can release a significant percentage of the daily IV dose during the first day but the dose goes below 10% in the long term. Therefore, in case of large antibiotic bone grafts implanted, it is recommended to set the systemic antibiotic dosing based on close monitoring of serum levels for a few days after surgery. Ciprofloxacin has to be monitored especially closely as it has the highest cytotoxic effect among the three drugs [44, 45]. A 10 mg/mL starting concentration of antibiotic was applied in our study and is also relevant according to the literature of local antibiotic drug release products regarding both amoxicillin [33, 46] and vancomycin [47]. We suggest that these calculations may have some relevance towards other antibiotic bone substitutes as well.

The release of the antibiotics was comparable or even faster when we used chitosan than in the absence of a polymer coating. Although Chi is a well-known vehicle for drugs, and it was already applied in combination with vancomycin, mostly with microencapsulation by spray drying [48–50], we did not find it effective in our experiments. This can be explained with our freeze-dried formulation and the solubility of chitosan. This polymer is only soluble in acidic media and the three chosen drugs were also acids or acidic salts. We speculate that the acidic drugs enhanced the solubility of Chi, since most antibiotics are also acids, and the use of chitosan as a delivery vehicle for sustained release is not suitable.

Ca-Alg was used as a layer by layer film coating, and in most cases alginate is used in loaded beads [51] or microspheres [52] or composites [53]. Since all the three drugs we used precipitated in the Na-Alg solution, we decided to use it as a coating to separate the drugs from water. The long-term release was successful when we produced a water insoluble Ca-Alg film coating. Due to the uneven surface of the bone structure, the thickness of the alginate film cannot be proven to be uniform on the surface of the bone. According to our current results this did not affect the elution characteristics. Although the coating exhibited little difference for amoxicillin, both ciprofloxacin and vancomycin proved to be suitable for sustained release bone graft formulations. The drug delivery period of at least 28 days should be sufficient for the long-term antibacterial effect required for the eradication of implant-related infections [54]. Loading biomaterials with antibiotic is nowadays a standard medical procedure for the local treatment or prevention of bacterial infection. However, there are concerns related to biofilm formation, developing resistance especially if the local antibiotic cement is the first line treatment [55]. One possible solution is that different antibiotics should be added locally than in the preventive phase or two or more antibiotics should be combined in local treatment [56]. Bacterial biofilm formation on bone cement has also been studied; adhesion to the bone cement is an important factor [57]. However, there is no evidence that an alginate coating on bone allograft would be suitable for biofilm formation. On the other hand, prevention of bacterial resistance can be solved with the use of combined drug coating later on in the* in vitro* experiments. Besides, we also have to keep in mind that both the allograft and the alginate coating are biodegradable, which can pose an obstruction for biofilm formation. According to our measurements, the MIC value of vancomycin was 0.2 *μ*g/mL for* Enterococcus faecalis* (data not shown) and the MIC value of 2 *μ*g/mL vancomycin is sufficient against vancomycin susceptible MRSA [58–60]. Taking into account that using our coating and vancomycin as the antibiotic we managed to keep the released vancomycin concentration above 5 *μ*g per mL at the 50th day, the* in vitro* results show that this drug coated biomaterial is capable of keeping the antibiotic above the required dose for a prolonged time.

## 5. Conclusion

We conclude that sustained release antibiotic bone graft coating can be achieved by using an insoluble Ca-Alg coating on bone allografts impregnated with antibiotics. This preparation allows the sustained release of either vancomycin or ciprofloxacin at therapeutic levels for at least 28 days, making this composition suitable for septic revision surgery. A short-term release coating without the protective alginate layer is also possible, especially with amoxicillin, a broad-spectrum antibiotic regularly used for the prevention of infection [33, 46].

## Figures and Tables

**Table 1:** Technical requirements against a local antibiotic formulation in the 3 main categories of orthopedic use in endoprosthesis surgery.

	Medical purpose	Probability of infection	Antibiogram	Required length of local antibiotic treatment	Typical local antibiotic formulation	Reference
Primary implantation	Prevention of infection arising from contamination at surgery or early postoperation	0.5–2%	Not available	1-2 days or until the surgical site is open through drainage	Antibiotic bone cement, off-the-shelf	[27–30]

Aseptic revision	Prevention of infection arising from either contamination or a low-grade infection	n/a	Not available, or its reliability is low	1-2 days or until the surgical site is open through drainage. Longer if low-grade infection is suspected.	Antibiotic bone cement, bone substitutes, freehand use of local antibiotic powder or solution	[27, 31]

Septic revision	Eradication of bacterial infection	100%	Available	Several weeks	Antibiotic bone cement, freehand use of local antibiotic powder or solution. Bone substitutes are contraindicated.	[18, 27, 31, 32]

**Table 2:** UV measurement characteristics of the investigated antibiotics.

	Characteristic absorbance (nm)	Linear absorbance-concentration interval
Amoxicillin	229	0.22–3.7
Ciprofloxacin	275	0.085–2.29
Vancomycin	280	0.06–2.00

**Table 3:** The daily released amount of amoxicillin measured with either replacing the solvent daily or replenishing the solvent. There were significant differences in the 1st and 2nd day with the different methods.

Released drug (mg/day)	Amoxicillin solvent replenishment	Amoxicillin cumulative release
	Mean ± SEM	Mean ± SEM
Day 1∗∗	0.260 ± 0.037	0.168 ± 0.030
Day 2∗∗∗	0.202 ± 0.026	0.098 ± 0.005
Day 3	0.081 ± 0.015	0.085 ± 0.016
Day 4	0.049 ± 0.013	0.063 ± 0.009
Day 5	0.032 ± 0.012	0.042 ± 0.012
Day 6	0.007 ± 0.002	0.021 ± 0.015
Day 7	0.003	0.001 ± 0.001
Day 8	0.003	0.002

**is the difference between the released drug on the first day with the two different methods, and ∗∗∗ is the difference between the released drug on the second day with the two different methods.

**Table 4:** Calculated antibiotic content of the coated bone compared to the daily doses in clinical practice.

	24 hours from 0.05 g bone	28th day from 0.05 g bone	24 hours from 45 g bone	28th day from 45 g bone	Daily dose/reference (mg/day)
Amoxicillin (mg)	0.26		234		6000 [33]
% of daily dose	0.004		3.9		

Ciprofloxacin (mg)	0.674	0.015	606.72	13.03	1500 [34]
% of daily dose	0.045	0.001	40.45	0.87	

Vancomycin (mg)	0.485	0.05	436.87	44.68	1000 [35]
% of daily dose	0.049	0.005	43.687	4.468	
